# Developing a Mobile App for Concussion to aid Patient Empowerment and Symptom Management

**DOI:** 10.7759/cureus.15972

**Published:** 2021-06-27

**Authors:** Janna Newton, Emily Wuerch, Noel Thomas, Boogyung Seo, Eddy Lang, Kiran Pohar Manhas

**Affiliations:** 1 Emergency Medicine, University of Calgary, Calgary, CAN; 2 Neurosciences, Alberta Health Services, Calgary, CAN

**Keywords:** concussion applications, concussion symptom management, patient empowerment, healthcare technology, adult concussion recovery, mild traumatic brain injury, mtbi

## Abstract

Despite the high prevalence of concussions each year in Canada, access to consistent and science-based information on how to self-manage these injuries remains a significant hurdle for many patients. Currently, available mobile applications (apps) focus mainly on supporting patients with sports-related concussions, although falls account for more traumatic brain injuries (TBI) than sports-related TBI’s in Alberta. Patients from a broader demographic may be limited from accessing information on how to correctly manage and track their symptoms as they feel that currently available resources are not applicable to them. Through collaboration between health system leaders, expert consultations, patients, and university students, a mobile app was designed as a platform to help patients manage and track symptoms at home, as well as to clarify misleading information and misconceptions surrounding injury. The team engaged numerous physicians, patient advisors, and health system leaders to improve upon the features of currently-existing concussion apps such as symptom tracking, insight into concussion, and strategies for returning to work/school that are more inclusive to adult, non-sports related injuries. We believe that these features will advance recovery by alleviating the burden of uncertainty and confusion for patients and their family members.

## Introduction

Concussion is a form of mild traumatic brain injury (mTBI) caused by an external force to the body, altering neurological function and characterized by a lack of anatomical lesions [1]. There are a wide range of symptoms associated with concussions, including headache, nausea, and impaired memory and attention. These symptoms typically last a few days up to 3-6 months, but can continue for a longer duration for some individuals [2,3]. Every year, 200,000 Canadians experience concussion [4]. Despite their prevalence, many patients report that there is a lack of resources available to them following a concussion, and that they are met with conflicting information online related to management strategies and what symptoms to expect [5]. Concussion management strategies are inconsistent, with no universally accepted clinical practice guidelines [6]. For example, online resources often recommend opposing management strategies: either stay in a dark room, or to return to normal life as soon as possible [7, 8]. This misinformation can result in some patients returning to the emergency department, unsure of how to manage their concussion [9]. Not only does this uncertainty burden the patients themselves, but also their support systems and the healthcare system as a whole. Many available resources target youth and athletes, although this comprises only a subset of concussion patients [10]. There are many differences in a non-sport adult population, such as the cause of injury and priorities for recovery (e.g. returning to work vs. returning to sport). As a result, there is a lack of clarity on management strategies, where patients with non-sports related injuries feel as if the existing resources are inapplicable to them. 

Our proposed solution is to develop a mobile app that promotes the self-management of adult, non-sports related concussions in partnership with a provincial health system: Alberta Health Services (AHS). The goal of this technical report is to outline the process that was undertaken to develop AHS Concussion Connection. This process has the potential to advance literature and be generalizable to additional publications. Market research was conducted to identify existing gaps in concussion management resources, and to determine what features were lacking in existing concussion apps. In addition, concussion patients, their families, and emergency medicine physicians were consulted for two purposes: (1) to identify the gaps in existing care and (2) to glean information on how this app could serve them and this community. Therefore, we have defined the “end-user” as our target population of patients, families and physicians. Lastly, we will describe the process of software and app development, and the future app objectives.

## Technical report

Input 

A provincial neurotechnology design competition brought together a multidisciplinary team consisting of university students, nurses, researchers, administrative leadership, and physicians. University students had backgrounds in software engineering, neuroscience, and kinesiology. Other expertise included emergency medicine, patient engagement, and health systems policy and planning, which first identified gaps in concussion care. This project was completed in collaboration with Alberta's multiple and unique Strategic Clinical Networks (SCNs), which aim to improve focused areas of healthcare by bringing patients and families, policymakers, clinicians and researchers together to collaboratively create change [11]. The Emergency Medicine SCN and Neurosciences, Rehabilitation & Vision (NRV) SCN were key to formulating change for concussion care in Alberta.

Materials

During development, the program Figma was used to demonstrate the user interface and outline the user experience. Figma was chosen given its ability to allow simultaneous collaboration amongst team members. Another advantage is that Figma is built with WebAssembly (WASM), allowing it to perform operations quickly [12]. Graphic designs for the app were created in Canva and PowerPoint. The team chose to build the app for iOS in the Swift programming language for efficiency, using the integrated development environment of Xcode. Further information regarding software programs used in app development can be found in Table 1.

**Table 1:** Outline of software terminology and programs utilized for app development IDE: Integrated Development Environment; MVVM: Model-View-ViewModel; API: Application Programming Interface

Terminology	Definition
Xcode	Integrated development environment built by Apple. It features support for languages like Swift and SwiftUI.
IDE	Software application that provides a suite of tools for software development.
Swift	Programming language widely used for the development of iPhone applications.
SwiftUI	Toolkit designed to augment the functionality of Swift and aids in the declarative development of Swift-based applications.
MVVM	Architectural design pattern for the development of mobile applications. Specifically, this separates the user interface and the business logic.
iCloud	Cloud storage and cloud computing service provided by Apple.
Core Data	Framework for that for data persistence and serialization for APIs.
API	Used to define an interface between different applications and software services.
Library	Collection of resources that help in software development.

Participants

Throughout the development process, patients, family members and emergency medicine physicians were recruited to share their insight and enable app co-design. Patients and families were recruited via the AHS patient advisor volunteer group. Emergency physicians from across Canada were recruited through health systems partners.

Process

**Figure 1:**
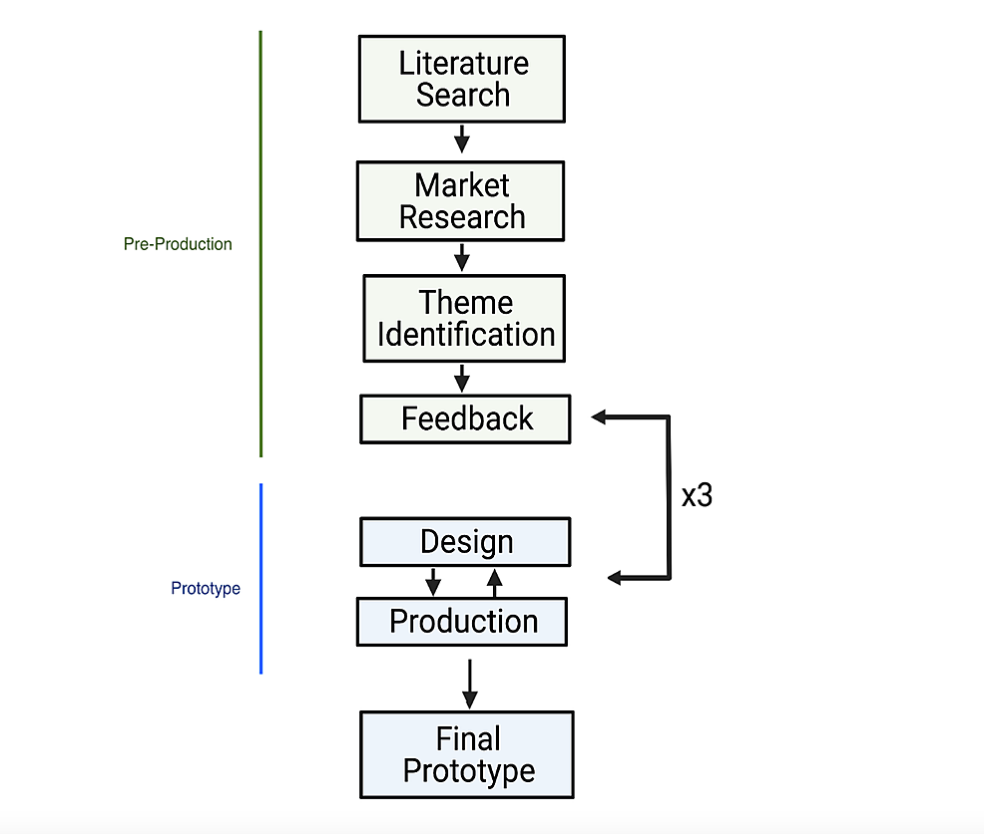
Application development process which includes feedback from patients, families, and physicians

An overview of the development process is described in Figure 1. Development began with a literature search, conducted using PubMed and Google Scholar. The following search terms were used to obtain information regarding current concussion management strategies: adult concussion management, concussion apps, mTBI and concussion care. The initial search demonstrated that current literature focuses heavily on sport-related concussions and youth. Further, concussion management strategies varied from an older, more conservative management strategy to the now more favoured active strategy. The newer approach consists of an initial 24-48 hour rest period before returning to activity, as opposed to a week or more of little to no activity [13].

Next, market research was conducted to investigate existing apps on Google Play and the App Store that aim to aid in concussion management. Ten apps were selected for thorough qualitative and quantitative review. Apps were evaluated based on the target population, rating and reviews on the app stores, quality of graphics, content, concussion assessment, management strategies, location services, and unique features. After a review of each criteria, a subjective assessment out of ten was given to the app according to the reviewer. From the information collected, the team was able to identify shortcomings of existing apps including narrow application to the concussion population (i.e., designed for children or sports-related concussions), no community resources, lack of symptom monitoring, and lack of descriptive information on how to return to work or school. 

An app outline was then created highlighting important features such as concussion information, symptom management, return to work timeline, and additional resources. This app outline was brought to health system partners as well as patient and family advisors for feedback. Patient interviews highlighted the importance of having a way to log and track symptoms, which can then be graphically represented to share with the care provider. Emergency medicine physicians recommended features such as a dark theme to reduce blue light exposure and having a visual summary of patient symptoms for ease of interpretation. After receiving feedback, the app was updated three times to reflect features which would simplify the cognitive load on end users. Content was explicitly evaluated by an emergency medicine physician to ensure validity of information. A final prototype was presented to patients and clinicians to get final feedback prior to pitching the app to field experts. 

**Figure 2:**
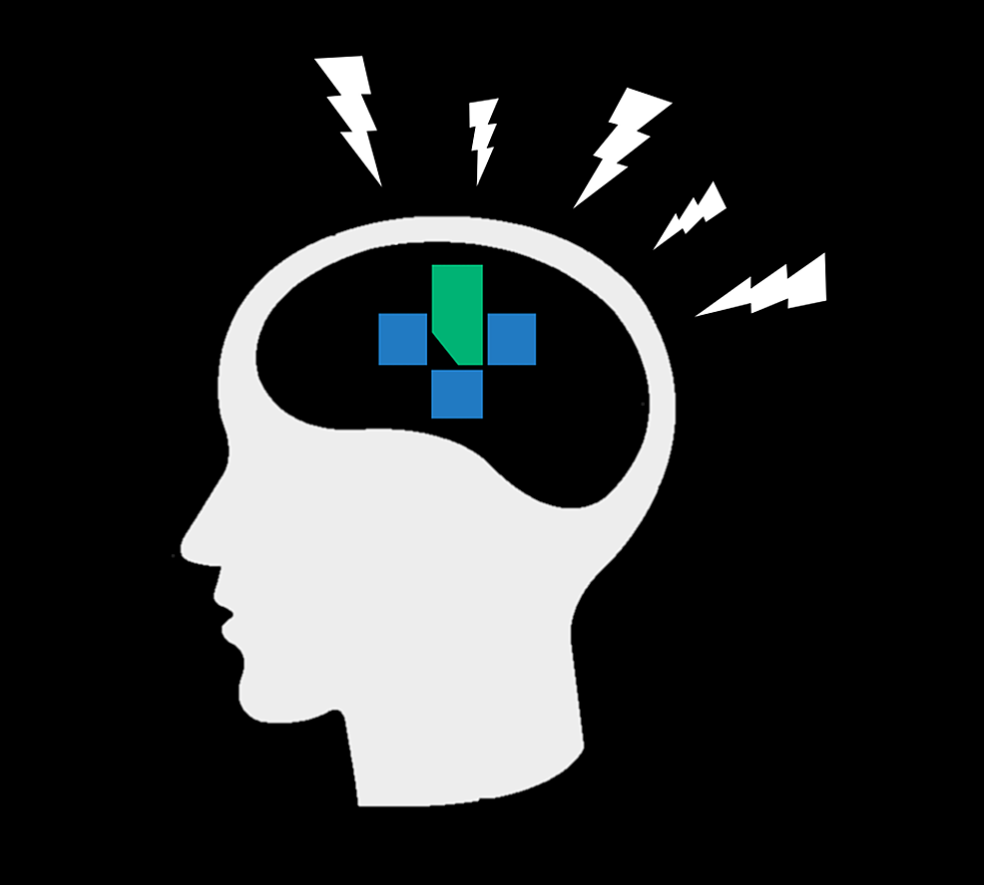
AHS Concussion Connection logo AHS: Alberta Health Services

Utilizing PowerPoint, marketing professionals, and logo design workshops, a simple logo for AHS Concussion Connection was created. The AHS Concussion Connection logo has the AHS logo (a blue medical cross that incorporates the shape of Alberta) within the brain to draw attention to both brain injury and Alberta’s health system. Lightning bolts surrounding the brain illustrates the neural injury sustained by the individual (Figure 2). A black background was chosen to be consistent with the dark theme of the app. This logo conveys the mission of supporting those who have experienced a concussion, and also reflects the support received from AHS.

App Development

Following pre-production and planning, a prototype of the app was developed. This development phase was further broken down into design and production (Figure 1). During the design phase, the goal was to identify target platforms, create an overview to map the required features, and determine the tools that would be used to implement these features. During production, features identified during the design phase would be translated into a usable product. First, a feasibility analysis was conducted to determine which features could be created by the team within a month. The second step was code implementation.

Design Phase

Platform Identification

Two factors were considered when identifying target platforms: feature requirements, and time constraints. The team chose to use SwiftUI, a programming language framework that supports rapid and responsive development. SwiftUI is built for iOS, the mobile operating system created by Apple; thus, the target platform chosen for the prototype was iOS.

Feature Determination

Here, a list of features that could aid in concussion management and should be included in the app was determined. This list was generated using information gathered in the pre-production stage. Throughout several rounds of feedback from healthcare experts and patient advisors, this list of features was modified accordingly. With each round of feedback, the list better reflected the needs of the patients.

Tool Identification 

Next, the team determined which tools would be most effective to create the prototype and implement the desired features. SwiftUI is used imperatively; it is difficult to know how the computer might interpret the code and will therefore slow down each testing cycle. In an effort to minimize this, an Xcode feature known as Canvas was used. This tool allowed developers to observe changes made in the code in real-time and ensure that compilation of the app happened less frequently [14]. Xcode also supports Core Data and iCloud, which is used to store information on the phone or in the Cloud, respectively; as the prototype matures into an app, this feature will be crucial. Thus, Xcode was the most appropriate choice of integrated development environment.

The version control system is used to manage different versions of the same project files. Git was chosen for this project, due to its robustness [15]. The team also implemented Github, a hosting service that manages Git repositories across multiple computers and several developers, enabling multiple team members to work on the files together with ease.

Production Phase

Feasibility Analysis

For each desired feature that was identified during the design phase, a feasibility analysis was performed. The team estimated how complex and time-intensive each feature was. This was done to ensure that the prototype could be completed in the time frame. If a feature was too demanding, the team either removed it from the list, or modified the feature to be more feasible.

Code Implementation

**Figure 3:**
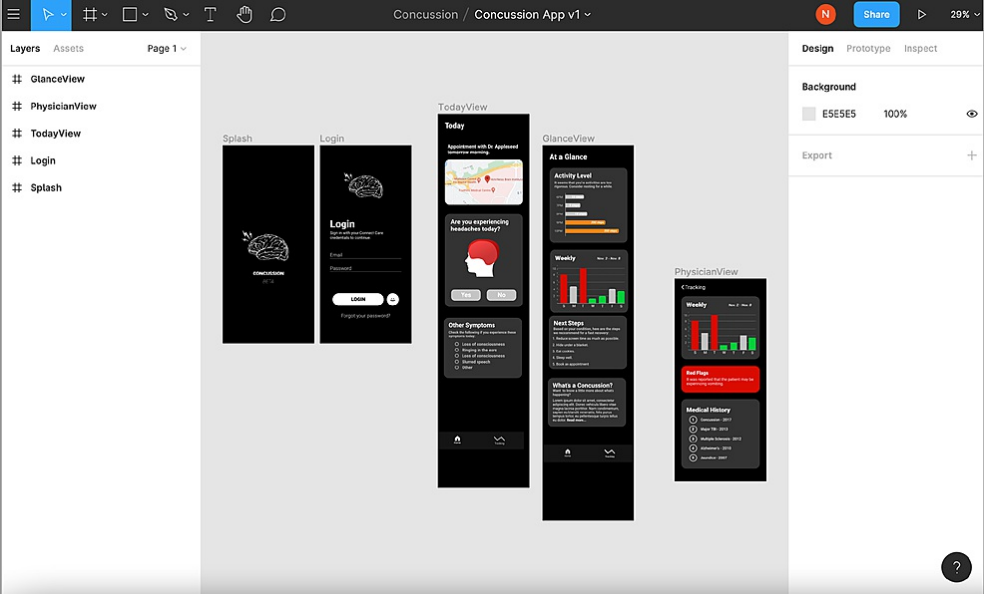
Figma design interface used to demonstrate the AHS Concussion Connection interface for end-users AHS: Alberta Health Services

Lastly, the features were implemented using the aforementioned tools and data. First, a skeleton or shell of the application was created, consisting of a login page, main menu, and connection to a NoSQL database. Here, the database is used as a placeholder, which will later be replaced with a connection to an application programming interface (API) as the application matures out of the prototype stage. This allows for the application to communicate with external resources. The application was split into several pages, all containing different features (Figure 3). Throughout development, pages and features were added and removed according to feedback. Several cycles of feedback and design editing occurred throughout the development of this prototype.

## Discussion

The impacts of concussion can be serious and long-lasting if not managed properly. Currently, patients do not have adequate tools to learn about concussion management and many of the existing resources are targeted towards athletes, including language and graphics that may not be inclusive to adult concussion patients. There is little treatment that can be done in the emergency department, leaving many patients confused as to what is the best course of action. A previous study demonstrated that patients recall 14% of spoken medical advice [16]. This issue is compounded by the fact that a common symptom of concussion is amnesia [17]. AHS Concussion Connection aims to improve management strategies for individuals that experience non-sports related concussions by providing them with information resources and helpful tools such as a symptom tracker. This app is unique from others on the market, as many existing apps focus on parameters such as when the individual can return to playing sports. 

This app was developed through a health system-trainee partnership, allowing students to learn from leaders in the healthcare system, and implement their expertise into a useful tool for the public. There are several advantages to an app developed from within the healthcare system, including the fact that all information is coming from a trusted source. Healthcare providers are in a unique situation to identify the gaps in care that currently exist, which could then be implemented into an application or other tool. This is a framework that could be used not just in the field of concussion, but across a variety of conditions.

This application has the potential to be expanded, not just across the province of Alberta, but across North America. This could be done in conjunction with the App Orchard, a marketplace of medical apps and health software commonly used by physicians and their patients. This app could be connected to an information sharing service, allowing physicians to have access to the current symptoms logged by their patients, as well as a patient history, including past concussions or head injuries. 

One potential limitation of this app relates to marketing and awareness, as individuals will likely not be aware of this app until after they experience a concussion. A potential solution is to connect physicians in emergency departments with this app; these physicians would then recommend this app to their patients. Another possibility is that, because this app is affiliated with AHS, individuals looking online for resources will identify our app as a source of quality information that can be trusted; this would help with the integration, as patients are sometimes bombarded with many different resources.

Another potential limitation pertains to challenges associated with screen time. Photo insensitivity (including computer/phone screen intolerance) are common symptoms of post-concussion syndrome [18], and concussion patients reported that limiting screen time was one of the most helpful modifications that aided in their recovery [19]. This may decrease the number of individuals that turn to apps for concussion management strategies. To combat this limitation, our app utilized a dark theme to reduce light exposure. We foresee that this app will be used for a short period of time each day, and will not likely result in long-term light exposure that may result from social media or video-streaming apps. 

Regardless of these limitations, we believe that AHS Concussion Connection contains all of the necessary features to become a useful tool for individuals that would like to improve the management of their concussion.

## Conclusions

Concussion research predominantly focuses on youth and sport-related concussions, thus missing a substantial proportion of concussion patients who require appropriate instruction and follow-up care. An app was successfully created to address the gap in care for adults who have sustained a non-sports related concussion, which can be expanded beyond a provincial level. Upon further development of the app, research should determine the impact in improving follow up care and return to work for patients who sustained a concussion.
